# A Simple Microwave Imaging System for Food Product Inspection through a Symmetry-Based Microwave Imaging Approach

**DOI:** 10.3390/s24010099

**Published:** 2023-12-24

**Authors:** Gennaro Bellizzi, Alessio Buzzin, Lorenzo Crocco, Antonio Mastrandrea, Noemi Zeni, Sabrina Zumbo, Marta Cavagnaro

**Affiliations:** 1 Department of Electrical Engineering and Information Technology, University of Naples Federico II, 80125 Naples, Italy; noemi.zeni@unina.it (N.Z.); sabrina.zumbo@unirc.it (S.Z.); 2 Department of Information Engineering, Electronics and Telecommunications, Sapienza University of Rome, 00184 Rome, Italy; alessio.buzzin@uniroma1.it (A.B.); antonio.mastrandrea@uniroma1.it (A.M.); marta.cavagnaro@uniroma1.it (M.C.); 3 Institute for Electromagnetic Sensing of the Environment, National Research Council of Italy, 80124 Naples, Italy; crocco.l@irea.cnr.it

**Keywords:** antipodal Vivaldi antenna, food inspection, microwave imaging, non-invasive diagnostic

## Abstract

In the food industry, there is a growing demand for cost-effective methods for the inline inspection of food items able to non-invasively detect small foreign bodies that may have contaminated the product during the production process. Microwave imaging may be a valid alternative to the existing technologies, thanks to its inherently low-cost and its capability of sensing low-density contaminants. In this paper, a simple microwave imaging system specifically designed to enable the inspection of a large variety of food products is presented. The system consists of two circularly loaded antipodal Vivaldi antennas with a very large operative band, from 1 to 15 GHz, thus allowing a suitable spatial resolution for different food products, from mostly fatty to high water-content foods. The antennas are arranged in such a way as to collect a signal that can be used to exploit a recently proposed real-time microwave imaging strategy, leveraging the inherent symmetries that usually characterize food items. The system is experimentally characterized, and the achieved results compare favorably with the design specifications and numerical simulations. Relying on these positive results, the first experimental proof of the effectiveness of the entire system is presented confirming its efficacy.

## 1. Introduction

In the food industry, products can be exposed to various contaminants that are potentially harmful to health. These contaminants include biological or chemical agents (such as bacteria, heavy metals, pesticides, and so forth) or solid contaminants like small fragments of glass, plastic, metal, wood, or other materials commonly used in packaging. Therefore, it is essential to monitor food items as they progress through the production chain to ensure their integrity before they reach the market [1,2]. Special attention is given to inspecting the presence of small foreign objects that may have entered the food during various production stages, especially during packaging [1].

This inspection can be performed offline on selected samples or directly in-line when the product moves along the production line. The former method is less efficient and more costly because the discovery of even a single contaminated item can lead to the disposal of an entire batch, and it still does not guarantee the detection of all contaminated pieces. Therefore, in-line inspection tends to be preferred.

Currently, several technologies are employed to perform this task: metal detectors, X-ray scanners, near-infrared sensors, or specialized optical cameras designed to seamlessly integrate into the production process [3,4,5,6]. However, there is still a notable rate of missed detections (false negatives). For example, metal detectors can only identify metallic objects, while X-ray technology may fall short in identifying low-density foreign materials, such as various types of plastics or fragile pieces of glass or wood. In addition, X-rays also pose potential risks to workers due to accidental exposure to radiation. Infrared sensors are limited by their penetration depth into the sample, and optical cameras are suitable only for transparent food and packaging. Hence, there is significant interest in exploring alternative solutions to address these limitations.

Recently, microwave imaging (MWI) [7] has emerged as an appealing alternative to conventional diagnostic tools [8,9,10,11,12]. This approach is entirely safe, using low-intensity non-ionizing radiation, and offers good penetration depth for packaged food, making it a cost-effective and easily integrable solution for industrial production lines [11]. Of course, MWI is only applicable to food items packaged in non-metallic containers, which nonetheless, represent a significant portion of cases in the food industry. These advantages have led to the recent development of dedicated MWI systems for food inspection [13,14,15].

In its basic configuration, an MWI system for food inspection consists of a pair of antennas positioned on both sides of the conveyor belt on the production line. The system probes the object under test (OUT) at different frequencies and at different positions of the OUT while it moves along the production line (when in proximity to the system) [11]. The antennas need to be wideband [16] to allow probing the OUT’s response with signals at sufficiently different frequencies, so to ensure adequate frequency diversity (i.e., to gather a sufficient amount of independent data) for reliable imaging. The antennas should even be ultrawideband (UWB) to guarantee an adequate imaging resolution for a large variety of products (from mostly fatty to high water-content foods), by exploiting different frequency sub-bands. It is worth noting here that the achievable spatial resolution is dictated by the wavelength, which in turn depends on the dielectric properties of the food. As such, different types of foods must be probed with different bands to ensure good imaging results in all cases without changes in the system (such a, e.g., antenna design).

While a great deal of work has been performed to design UWB antennas to be used in several MWI applications, in particular, for the most investigated one, i.e., breast cancer detection (e.g., [17,18,19]), to the authors’ knowledge, until recently no UWB design was specifically devoted to food inspection. To address this point, the design of a circularly loaded antipodal Vivaldi antenna (CLAVA) was recently proposed [14]. This antenna was characterized by a broad operational band, from 1 to 15 GHz and was able to meet the requirements of compactness and cost-effectiveness, being a printed antenna.

Regarding the image formation approach, the key requirements are a negligible computational burden to enable real-time imaging and a sufficient sensitivity, to reliably image even small foreign bodies, such as millimeter-sized inclusions. To achieve these features, the first proposed solution (hereafter referred to as the reference-based (RB) approach) is a differential approach where the imaging is performed by processing the difference between the data acquired for the OUT and those of a contaminant-free object (acquired once at the beginning and stored in memory) used as a reference [11]. However, this solution has the inconvenience that the reference object may be not identical to the OUT, or the positions at which the OUT is probed may not exactly match those at which the data of the reference object are acquired. As a result, a significant non-null differential signal may occur even in the absence of inclusions, thereby leading to a possibly high rate of false positives. To avoid using a reference object, an alternative approach, hereafter referred to as the symmetry-based (SB) MWI approach, has been recently proposed [20]. SB-MWI is still a differential approach; however, the reference data are obtained from the data of the OUT itself, by exploiting its inherent “symmetries” (a typical example of symmetric OUT is a product packaged in a circular jar). The idea underlying the approach is that if the overall system, the OUT plus the antenna system, exhibits certain spatial symmetries, namely does not change for rigid spatial transformations (like rotation, mirroring, and so on), the same happens to the acquired data, provided they are collected consistently with the considered symmetry. Therefore, as long as no inclusion is present, the difference between the “original” and “transformed” data is zero. Conversely, if an inclusion is present and its position in the OUT is such as to disrupt the symmetry, the acquired data are no longer symmetric, and the difference between the original and transformed data is no longer zero. This difference can then be used to detect and image the inclusion. Of course, this approach works solely in the case of symmetric food items, which, however, is a common feature in practice.

Considering the above, the aim of this paper is to present a simple MWI system for in-line food monitoring that exploits a pair of properly arranged CLAVAs and the SB approach to form the image. To this end, a comprehensive characterization of the CLAVA is performed, comparing the realized antenna with the designed one. Additionally, a proof-of-concept experiment is presented, to prove the effectiveness of an MWI system equipped with the specified antennas and the SB approach in simplified conditions.

The paper is organized as follows: Section 2 provides an overview of the design of CLAVA, of the basic architecture of the MWI system, and of the SB imaging approach. Then, the experimental setup (including the MWI system), built to carry out the imaging experiment, is presented. Section 3 shows and discusses the results of both the experimental characterization of the antenna system and the imaging experiment carried out by using the system and the SB approach. Conclusions follow in Section 4.

## 2. Materials and Methods

### 2.1. Antenna Design

To implement an MWI device that can maintain consistent performance standards regardless of the specific food being inspected (i.e., irrespective of varying dielectric properties), the use of a UWB antenna is mandatory [21,22]. To achieve this goal, a frequency range of operation spanning from 1 GHz to 15 GHz has been considered. This range strikes an optimal balance between the achievable spatial resolution and the ability to penetrate diverse types of food materials. Furthermore, it is essential that the designed antenna meets criteria such as compactness, affordability, and resilience in the presence of other antennas within the MWI array. In this respect, the WR187-horn antenna, considered in the preliminary numerical study [20] does not meet the above requirements because of its large size and restricted bandwidth (of only 2 GHz, from 3.95 to 5.85 GHz). Therefore, for the present study, a new antenna was designed.

**Figure 1:**
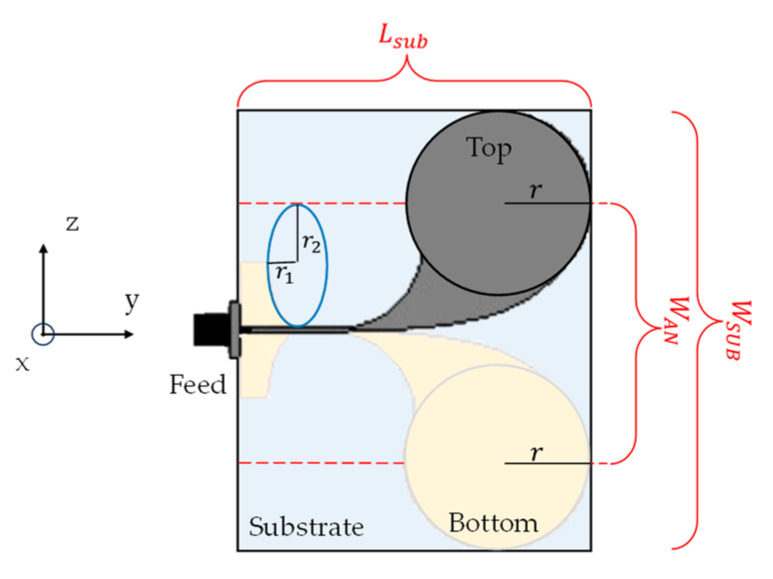
Sketch of the layout of the designed antenna (top view).

The chosen antenna design adheres to the typical antipodal Vivaldi antenna configuration, enhanced by the incorporation of circular loads, referred to as CLAVA [14,19] (see Figure 1 for reference). The circular loads serve the dual purpose of extending the lower operating frequency range and enhancing the antenna’s radiation characteristics, while maintaining a compact antenna size. In fact, they represent a capacitive load for the traditional Vivaldi design, which reduces the resonant length of the antenna, thus allowing for a decrease in the lowest frequency of operation while maintaining the same antenna dimensions.

Additionally, it is worth noting that the Vivaldi antenna is well-regarded for its directional properties, which are crucial for effectively radiating objects positioned just a few centimeters in front of the antenna.

The design methodology for the antipodal Vivaldi antenna was adapted from [21], with modifications to the dimensions and structure to better meet the specific requirements at hand. For the actual design of the CLAVA, after constructing the antenna as described in [21], an optimization process was undertaken to reduce the antenna dimensions, in order to enable a minimal impact on the structure of the production line. To this end, the first strategy was to choose a substrate dielectric material with high permittivity. In particular, RT/Duroid 6010LM was chosen, which has a dielectric constant of *ε_r_* = 10.7, loss tangent *τ* = 0.0023, thickness *h* = 0.0635 mm, respectively; the copper thickness from which the antenna is built is *t* = 0.017 mm.

The selected dimensions for the Vivaldi antenna must obey with the following equations [21]:

(1) $$L_{sub}=\frac{c}{f_{L}}\sqrt{\frac{2}{\varepsilon_{r}+1}}$$

and

(2) $$W_{AN}=\frac{c}{2f_{L}}\sqrt{\frac{2}{\varepsilon_{r}}}$$

where *L_sub_* and *W_AN_* are, respectively the length of the antenna and the opening of the flare (Figure 1), *c* is the speed of light, *f_L_* is the lowest operating frequency and *ε_r_* the dielectric permittivity of the material used for the substrate. 

As mentioned above, the desired lower cutoff frequency is 1 GHz and the relative dielectric permittivity of the substrate is *ε_r_* = 10.7. Substituting these values in (1) and (2), we obtain *L_sub_* = 124 mm and *W_AN_* = 45.85 mm. However, these dimensions still exceed the requirements for the intended application, making it incompatible with the fundamental design criterion: antenna compactness. To address this challenge and balance antenna size and bandwidth, two circular antipodal loads were introduced onto the radiator and ground, as illustrated in Figure 1, with radius *r* = 14.78 mm. This approach enables sustaining the desired bandwidth while reducing the antenna’s overall size [14].

The antenna parameters were optimized by way of simulations performed using CST Studio Suite (Dassault Systèmes, Vélizy-Villacoublay, France). The parameters considered in the optimization process were the substrate length *L_sub_* and width *W_sub_*, which define the antenna’s overall dimensions and affect its matching. 

Additionally, the input microstrip line width *W_f_* as well as the curvature of the ground plane to allow the transition from the input microstrip line to the balanced line feeding the antenna were also optimized. The final design foresees antenna dimensions of *L_sub_* = 50 mm and *W_sub_* = 71.56 mm; a microstrip width of *W_f_* = 0.55 mm, and a curvature of the lower ground section realized through a quarter ellipse with semi-axes equal to *r*_1_ = 5.7 mm and *r*_2_ = 13.725 mm (refer to bottom part of Figure 1).

Other details about the antenna design and the parameters optimization can be found in [14]. Two prototypes of the CLAVA antenna were realized using the photolithography process, to allow for the performance of the MWI experiments.

### 2.2. Basic Structure of the MWI System and Measurement Scheme

**Figure 2:**
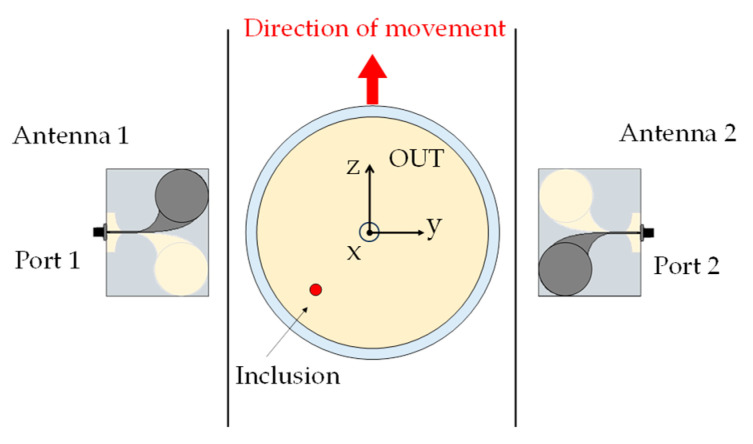
Sketch of the overall MWI system, OUT plus antenna system (top view).

The basic architecture of the MWI system is shown in Figure 2. As anticipated, it consists of two CLAVAs positioned in front of each other on both sides of the production line. Each antenna works as both a transmitter and a receiver allowing one to acquire the 2 × 2 scattering matrix, *S_ij_ i,j* = 1, 2, by using a two-port vector network analyzer (VNA). To increase the number of independent data available, the *S_ij_* are acquired at 2M + 1 different positions, *z*_±_*_m_* = ±*m*Δ_z_, *m* = 0, …, M, assumed by the OUT when approaching the system, having assumed as z_0_ = 0 cm the position of the OUT when it is in the middle between the two antennas (as shown in Figure 2). Moreover, for each probing position, the *S_ij_* are measured at N frequencies, say *f_n_*, *n* = 1, …, N (for further details, the reader is referred to [20]).

### 2.3. MWI Approach

As stressed in the introduction, the adopted SB approach was first presented in [20]. It exploits the symmetries that typically characterize the OUT and the MWI system, thus avoiding the need for additional reference measurements as in the RB approach [11].

**Figure 3:**
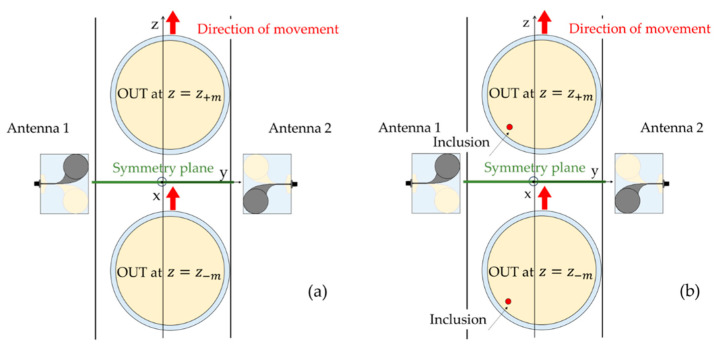
Schematic representation of the possible symmetries of the system. The system is symmetric with respect to the xy-plane: (**a**) the system, when the OUT occupies the position *z_−m_*, is specular to the system when the OUT occupies the position *z_+m_*; (**b**) the presence of an inclusion impairs the symmetry.

A typical case (referred in [20] to as the orthogonal symmetry plane (OSP)-based approach) is depicted in Figure 3a, where the OUT has circular symmetry (a quite common case in the food industry) and each antenna is characterized by its own symmetry plane (the xy-plane in figure), which virtually splits the antenna into two identical halves (this is quite common in antenna design, including the developed CLAVA, for which the symmetry plane is the xy-plane of Figure 1). In this case, if the two antennas are placed in front of each other with their symmetry planes coincident and orthogonal to the direction of motion (as shown in Figure 3a, where the symmetry planes coincide with the xy-plane), the overall system, observed when the OUT is at *z* = *z_−m_*, appears perfectly specular to the overall system observed when the OUT is at *z* = *z_+m_* (*m* = 1, …, M). As a result, the transmission parameters, *S*_21_ and *S*_12_, measured by the couple of antennas, when the OUT is, respectively at *z* = *z_−m_* and at *z* = *z_+m_*, are equal, so that their difference is zero (below the measurement noise, in practice). However, if an inclusion is present in the OUT (see Figure 3b), such symmetry is lost, and the transmission parameters are no longer equal. 

Then, their difference can be used to detect and image the inclusion (for more details about the inversion procedure, the reader is referred to [20] as well as to Appendix A).

As pointed out in [20], the advantage of this approach is that the symmetry is preserved even when the OUT is not evenly spaced from the two antennas, but is slightly displaced towards one of them, as shown in Figure 3a. This happens because the displacement is along the symmetry plane. Another advantage is the use of the transmission parameters as the input data of the inversion. Indeed, thanks the reciprocity, *S*_21_ = *S*_12_, the antennas are exchangeable. This implies that the antennas can be even different from each other, provided that they have a same symmetry plane. A drawback of this approach is the limited number of the data that is exploited (only the measured transmission parameters) which results in the imaging of the contaminant not being unique (as it will be shown in Section 3.2). 

It is worth noting that, in addition to the SB approach described above, another approach was proposed in [20], referred to as the parallel symmetry plane (PSP)-based approach, applicable when the overall system also exhibits a symmetry “left-right”, i.e., it is also symmetric (i.e., specular) with respect to the plane parallel to the direction of motion (xz-plane in Figure 3). Indeed, the combined use of both symmetries has the advantage of increasing the number of input data to be processed and of improving the imaging performance with the consequent addressing of the above ambiguity in the imaging of the inclusion [20]. Unfortunately, this latter approach is more sensitive than the former to errors such as antenna mismatch, antenna misalignment, the OUT not being perfectly centered between the two antennas, and so on, therefore its exploitation requires the more precise and accurate assembly of the system. Accordingly, the SB approach (i.e., the OSP-based approach in [20]) is easier to implement in practice, more robust to the inaccuracies of the experimental system, therefore, it will be the symmetry assessed in this paper through the implemented MWI system.

As a concluding remark, it is worth mentioning that MWI approaches exploiting symmetries have been already proposed for the imaging of brain stroke, leveraging the supposed “left-right” symmetry of the human head [23]. In any case, the approach in [23] is similar (with some relevant differences) to the PSP-based approach, but not to the OSP-based approach applied in this study.

### 2.4. Numerical Green’s Function

As described in [20], the SB images the inclusion from the data by solving a linear inverse problem, where Green’s function is the dot product between the electric fields radiated by the two antennas in the OUT (without the inclusion), evaluated at each frequency, *f_n_*, and position, *z_±__m_*. Therefore, one needs to know Green’s function to reconstruct the inclusion from the measured data. A way to do this is by numerical simulations. Again, we have employed the CST Microwave Studio. 

**Figure 4:**
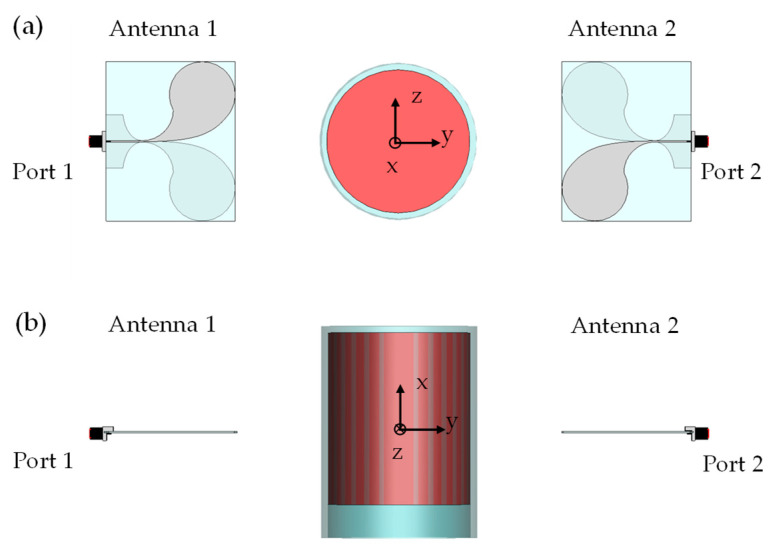
Numerical model of the overall system in the CST Microwave Studio environment: (**a**) top view; (**b**) front view.

The adopted numerical model is depicted in Figure 4 (in the case of OUT at the position *z* = 0 cm). As in [20], Green’s function was computed by placing the OUT at each of the 2M + 1positions, *z_±m_* = ±*m*Δ*_z_* (*m* = 0, …, M, M = 6 and Δ*_z_* = 1 cm) and running the simulation, over the frequency band 4–7 GHz (this is the sub-band exploited in Section 3 for the imaging). The data vectors, of N*P elements, extracted from each simulation (being N = 13 the number of evenly spaced frequency samples, over the range 4–7 GHz, and P = 84,054 the number of cells of the mesh in the OUT), were then organized in a rectangular matrix, of size M*N × P, relative only to the positions *z_+m_* with *m* = 1, …, M, representing Green’s function used in the SB approach (for more details about the inversion procedure, the reader is referred to [20]).

**Figure 5:**
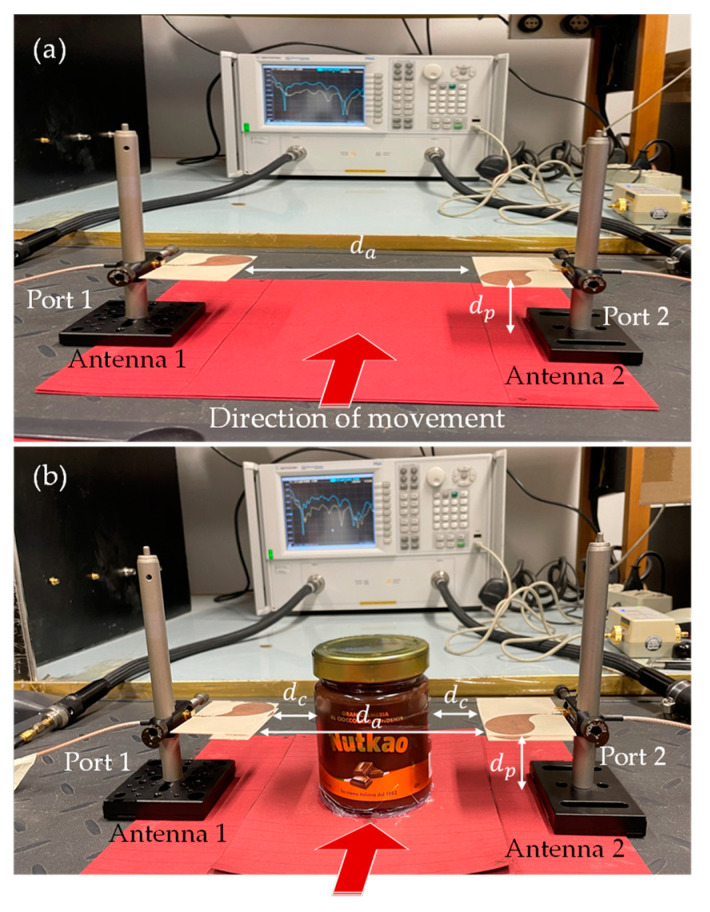
Experimental setup: the two CLAVAs facing each other without (**a**) and with (**b**) the OUT placed in the middle. The red arrow indicates the direction of the OUT movement.

In conclusion, it is worth noting that, while the considered model is realistic, it does not perfectly reproduce the experimental setup (see Figure 5b). Indeed, while the antennas are accurately modeled, all the other parts, such as the cable connecting the antennas to the VNA, the antenna supports, and the system’s workbench, are either roughly recreated or even missing in the model. Even the OUT is not perfectly modeled.

Indeed, while the shape and sizes are quite faithful to the actual OUT, the food level in the jar, the jar thickness (assumed to be uniform and equal to 3 mm in the model) and so on, are not accurately reproduced. The values of the complex relative permittivity of the different materials composing the OUT, here assumed equal to 6 for glass and equal to *3 − j* 0.42 for cream, may also be different over the whole analyzed frequency range. Indeed, these values are the ones measured for the product investigated in [11] and are averaged over the investigated frequency range. Of course, an imprecise numerical model such as ours translates into an inaccurate computation of Green’s function and so to a worsening of imaging performance. However, more accurate modelling is hard to achieve in practice due to the increase of the uncertainty associated with a greater number of involved parameters (both electromagnetic and geometrical). Nonetheless, a less accurate model was intentionally employed in order to test the robustness of the SB approach against the model’s uncertainties.

### 2.5. Measurement Setup

Figure 5 shows a picture of the realized MWI system. According to the scheme in Figure 2, the system comprises two CLAVAs placed in front of each other at a distance *d_a_* = 15 cm (Figure 5a). The antennas are mounted parallel to the bench on which the overall system is placed, at a height *d_p_* = *5* cm. To exploit the SB approach, the antennas are aligned with their symmetry planes orthogonal to the direction along which the OUT moves (red arrow in Figure 5a).

The data (scattering parameters *S_ij_* (*i,j* = 1, 2) are measured by two VNA ports (PNA Network Analyzer E8363C, Agilent Technologies (Santa Clara, CA, USA, [10 MHz–40 GHz]) connected to the antennas through two coaxial cables.

To ensure the symmetry of the overall system, a (homogeneous) chocolate-hazelnut cream packaged in a circular jar of glass (radius of 4 cm and height of 10 cm) was chosen as the OUT. Figure 5b shows the system with the OUT positioned in the middle between the two antennas (at the distance *d_c_* = 4 cm from each of them).

**Figure 6:**
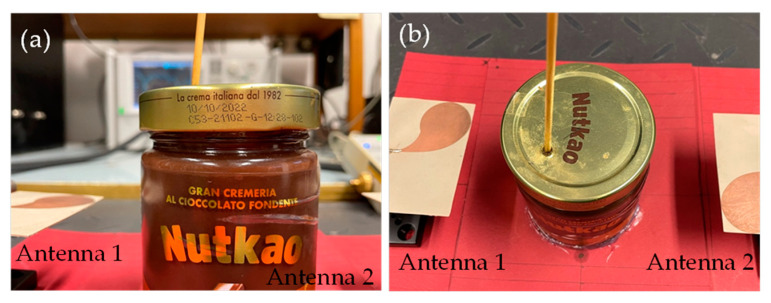
Positioning of the metallic inclusion inside the OUT: (**a**) front view; (**b**) top view.

The presence of an inclusion in the OUT was simulated by inserting a metallic sphere, 1 cm in size, in the cream positioned at a height of about 5 cm in the jar, on the left-bottom quadrant (see Figure 6). Note that the size and the material of the inclusion are not ones that are typically encountered in practice, where the contaminant is usually smaller (around 5 mm in size), and most undetected contaminants are non-metallic (such as glass, wood, or plastic splinters). Nevertheless, this configuration is valid for our main purpose, namely, to provide the first experimental proof of the working principle of the SB approach [20], in a simple, highly inaccurate experimental set-up.

As a matter of fact, despite its low complexity, several inaccuracies may have occurred in the implementation of the set-up, due to, e.g., the differences in the realization of the two CLAVAs, their manual positioning, and the OUT movement at the different *z_±m_* positions along the line. Of course, a significant improvement in the performance is expected with a more complex and accurately implemented/assembled system.

In any case, a preliminary estimate of the type and minimum size of the inclusion that can be effectively detected was provided in the numerical study in [20].

The imaging experiments were carried out with and without the inclusion in the OUT in order to enable a comparison with a control case.

As already pointed out, the movement of the OUT on the production line was reproduced by manually moving it along the direction of the red arrow in Figure 5b, corresponding to the z-axis in Figure 2. The data were acquired at different positions of the OUT, ranging from −6 cm to +6 cm, with a step of 1 cm (i.e., *z_±m_* = ±*m*Δ_z_, with *m* = 0, …, M, M = 6 and Δ_z_ = 1 cm). Note that this number of measurements is consistent with the constraints arising from the typical speed of production lines and VNA acquisition rates [11]. The reference position, *z*_0_ = 0 cm, is when the OUT is in the middle between the two antennas (see Figure 5b). For each position, the data at N = 13 frequencies, ranging from 4 to 7 GHz with a frequency step of 0.25 GHz were acquired. It is worth noting that, while the antennas are characterized in a wider band, from 1 to 15 GHz as shown in Section 3, only a sub-band was used in the imaging experiments. This choice is in part motivated by the need to limit the detrimental effects the imperfect manual alignment of the antennas on the symmetry, as well as the imperfect manual positioning of the OUT at the specular positions *z*_±*m*_, which are surely more pronounced at higher frequencies. In addition, this choice still allows us to treat the inclusion as a small scatterer and so to face the imaging problem at hand as a linear inversion problem (although the inclusion is surely not a weak scatterer).

### 2.6. Measurement Procedure

The measurement procedure begins with the full two-port calibration of the VNA. As previously reported, the considered frequency range is 4–10 GHz. 1601 frequency samples were acquired, with an IF bandwidth of 1 kHz, and an incident power set to 0 dBm.

Concerning the characterization of the antennas, two sets of measurements were carried out. First, the reflection coefficient at the input port of each antenna was measured with the antenna standing alone. This was performed to evaluate whether the measured bandwidth (at −10 dB) matches the design specifications and the simulation results. Then, the four scattering parameters of the antenna system (see Figure 5a) were measured to assess how the reflection coefficients of the antennas change and to evaluate the levels of the transmission parameters, *S*_12_ and *S*_21_, which are the parameters exploited for the imaging.

Regarding the assessment of the SB approach, as already stressed, two different imaging experiments were carried out (according to the measurement scheme described in Section 2.2 and Section 2.3):

- OUT with the inclusion inside.
- OUT without the inclusion.

The latter serves as control case, to check that in the absence of an inclusion, no image is produced by the SB approach. In addition, these data are used as reference data to implement the RB approach, to allow a comparison with the reconstructions achieved by the SB approach.

## 3. Results and Discussion 

### 3.1. Antenna Performance

**Figure 7:**
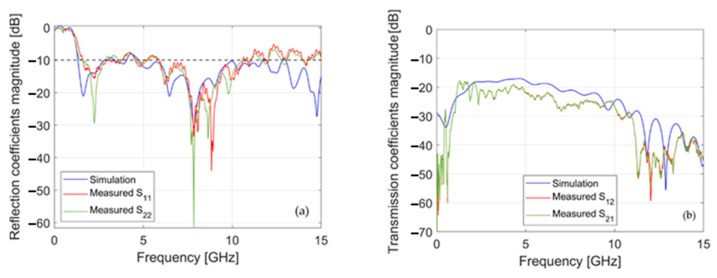
Comparison of the measured scattering parameters with those simulated in [14]: (**a**) reflection coefficients magnitude vs. frequency; (**b**) transmission coefficients magnitude vs. frequency.

Given that the foreseen application of the designed CLAVA is in the near field of the antenna, with two elements that face each other (see Figure 5), the main parameters of interest are related to the scattering coefficients. Figure 7 shows the scattering coefficients of the numerically designed antenna compared with those measured from the two realized prototypes. In particular, Figure 7a shows the reflection coefficient magnitude, while Figure 7b reports the transmission coefficient. The measurements were carried out with the two antennas placed 15 cm apart from each other, in the absence of any object in between, in a noisy environment, in accordance with what was mentioned in Section 2.5 (see Figure 5). From Figure 7 a good agreement can be derived between the simulations and the measurements. At the same time, it can be noted that, due to the limited accuracy of the manufacturing process, the two antennas have several differences, both between the two prototypes and with the simulated data, which nonetheless do not invalidate the matching nor the transmission of the signal. Considering the transmission coefficient, the lower measured values indicate the efficiency of the two antennas, with particular reference to their losses. Still, considering the transmission coefficient, the absolute values (both simulated and measured) indicate the ability of the antennas to focalize the near field in front of them. 

**Figure 8:**
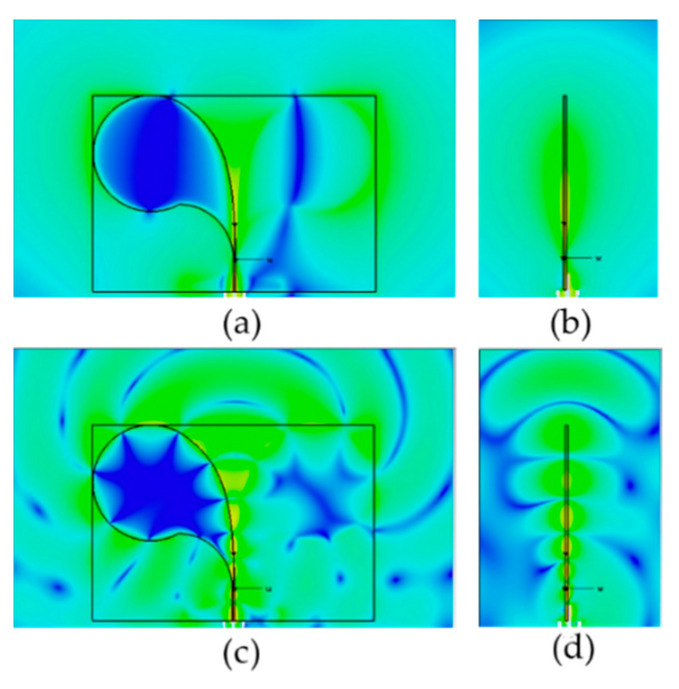
Electric field distribution near the CLAVA antenna at 2 GHz (**a**,**b**) and at 10 GHz (**c**,**d**) on the E-plane (**a**,**c**) and on the H-plane (**b**,**d**). Color scale in dB_max_ between 0 dB and −80 dB.

Figure 8 reports the instantaneous (phase 0) electric field distribution near the considered antenna in two orthogonal planes at the two frequencies of 2 GHz and 10 GHz, i.e., close to the lowest and the highest frequencies of operation. From the figure, the end-fire near-field operation can be appreciated. 

**Figure 9:**
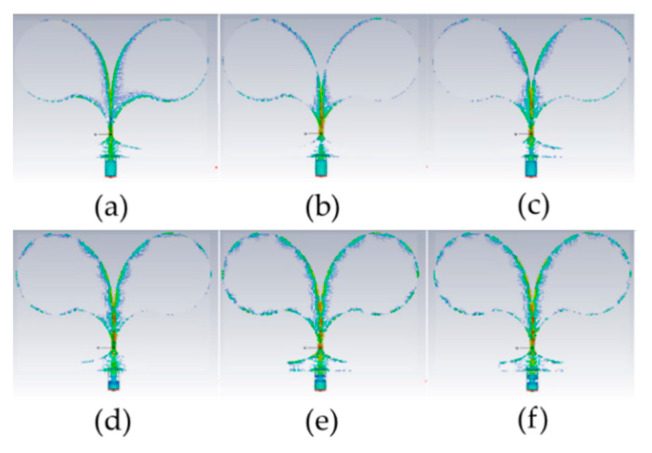
Current density distribution on the CLAVA antenna at different frequencies in the frequency band of operation, in dB_max_. Color scale between 0 dB and −40 dB. (**a**) 1 GHz; (**b**) 2 GHz; (**c**) 3 GHz; (**d**) 5 GHz; (**e**) 10 GHz. (**f**) 15 GHz.

Similarly, Figure 9 shows the instantaneous current density distribution (phase 0) on the CLAVA antenna at different frequencies within the band of operation. From the figure, the role of the circular loads as well as of the smooth transition between the input microstrip line and the coplanar line making the Vivaldi radiator is evident.

Finally, Table 1 reports a comparison of some UWB antennas proposed in the recent literature for different applications. From the table, it is evident how the addition of the circular loads allows for a reduction in antenna dimensions while maintaining quite a low minimum frequency of operation.

### 3.2. Assessment of the SB Approach

**Figure 10:**
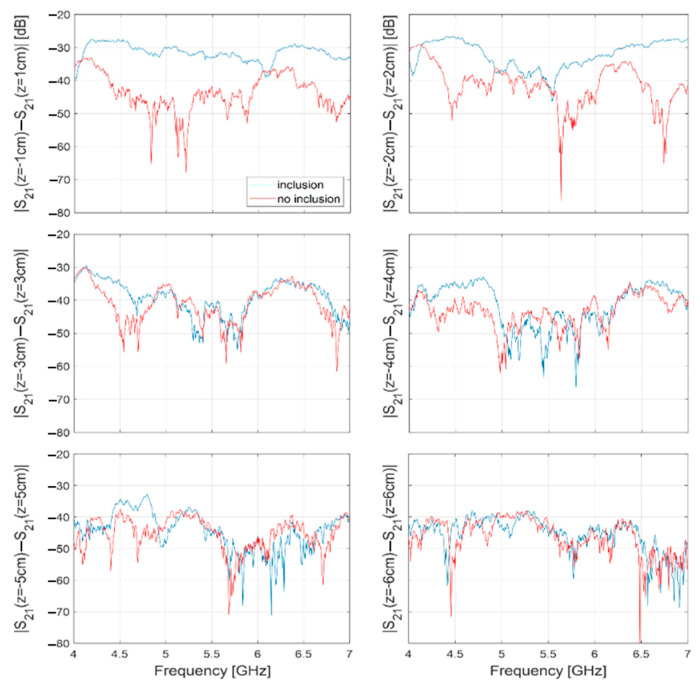
Magnitude (in dB), vs. frequency, of the difference between the transmission parameters measured when the OUT is at *z_±m_* = ±1, ±2, …, ±6 cm. (blue lines: OUT with inclusion; red lines: OUT without inclusion).

Figure 10 shows the magnitude (in dB), vs. frequency, of the difference *S*_21_(*z*_−*m*_, *f*) − *S*_21_(*z*_+*m*_, *f*), measured when the OUT is positioned at *z*_±*m*_ = ±1, ±2, …, ±6 cm along the z-axis (it is worth recalling that the antennas are mounted with their symmetry planes coincident with the xy-plane, i.e., with the plane of equation *z* = 0, so that the probing positions, *z*_±*m*_, are specular to each other with respect to such plane). Specifically, the blue lines represent the data relative to the OUT with inclusion and are the data provided in input to the inversion algorithm implementing the SB approach. For comparison, Figure 10 also shows the same data, but relative to the OUT free of the inclusion (red lines).

Note that the figure provides the difference over the frequency range 4–7 GHz, in agreement with what stated in Section 2.3.

From Figure 10, it can be noted that the blue lines dominate over the red lines. Since |*S*_21_(*z*_−*m*_, *f*) − *S*_21_(*z*_+*m*_, *f*)| can be interpreted as an indicator of the degree of symmetry of the OUT (the larger its value the lower the degree of symmetry of the OUT), this implies that in the case of the blue data, the OUT exhibits a reduced symmetry, in agreement with the presence of the inclusion. Therefore, the presence of the inclusion impairs the symmetry of the system, resulting in a remarkable difference between the transmission parameters measured at specular positions, *z*_±*m*_, of the OUT along the line.

Furthermore, note that a non-null |*S*_21_(*z*_−*m*_, *f*) − *S*_21_(*z*_+*m*_, *f*)| (i.e., above the noise level, but not larger than about −35 dB) is also observed in the case of the OUT free of the inclusion. This suggests that the overall system is not perfectly symmetrical even when no inclusion is present in the OUT, confirming the expectations of the need to improve the accuracy with which the system is implemented.

Such a difference can arise due to several factors, including misalignment between the two antennas or between the antennas and the direction of movement, or the inaccurate positioning of the OUT at the specular positions *z*_±*m*_. However, such a difference is notably lower, by up to 20 dB, than the difference observed in the presence of the inclusion (as in the case *z*_±*m*_ = ±1 cm). Moreover, it can be further reduced by removing (or mitigating) the sources of “asymmetry” mentioned above. This is an encouraging outcome, especially in view of the practical applications of the method, where the goal is to detect and image smaller and less contrasting inclusions. 

From Figure 10 it can be also noted that the discrepancy between blue and red lines is more pronounced in the case *z*_±*m*_ = ±1, ±2 cm, gradually reducing in the other cases. This trend is coherent with the fact that at *z*_±*m*_ = ±1, ±2 cm the OUT, hence the inclusion, is closer to (and more in front of) the antennas than the other positions, thus yielding a more intense scattered signal. As a matter of fact, over the band 4–5 GHz, the discrepancy between the blue and red lines in the case *z*_±*m*_ = ±4 cm is slightly larger the one in the case *z*_±*m*_ = ±3 cm, which, in turn, is slightly larger the one in the case *z*_±*m*_ = ± 5 cm. This behavior may be an effect of the inaccurate positioning of the OUT at the specular positions *z*_±*m*_, which, as pointed out in Section 2.5, was carried out manually in our experiments. Possibly, in the results in Figure 10, the positioning at *z*_±*m*_ = ±3 cm, in the case of the OUT without the inclusion, was accidentally less precise than the positioning at *z*_±*m*_ = ±4 cm, thus determining a higher level of the red line and so a smaller difference from the corresponding blue line. On the other hand, this explanation seems to be confirmed by the results relative to *z*_±*m*_ = ±6 cm where no remarkable difference is observed between the red and the blue lines, indicating that in this case the positioning error has no remarkable effect, consistent with the fact that at such positions, the OUT is beyond the antennas’ aperture.

**Figure 11:**
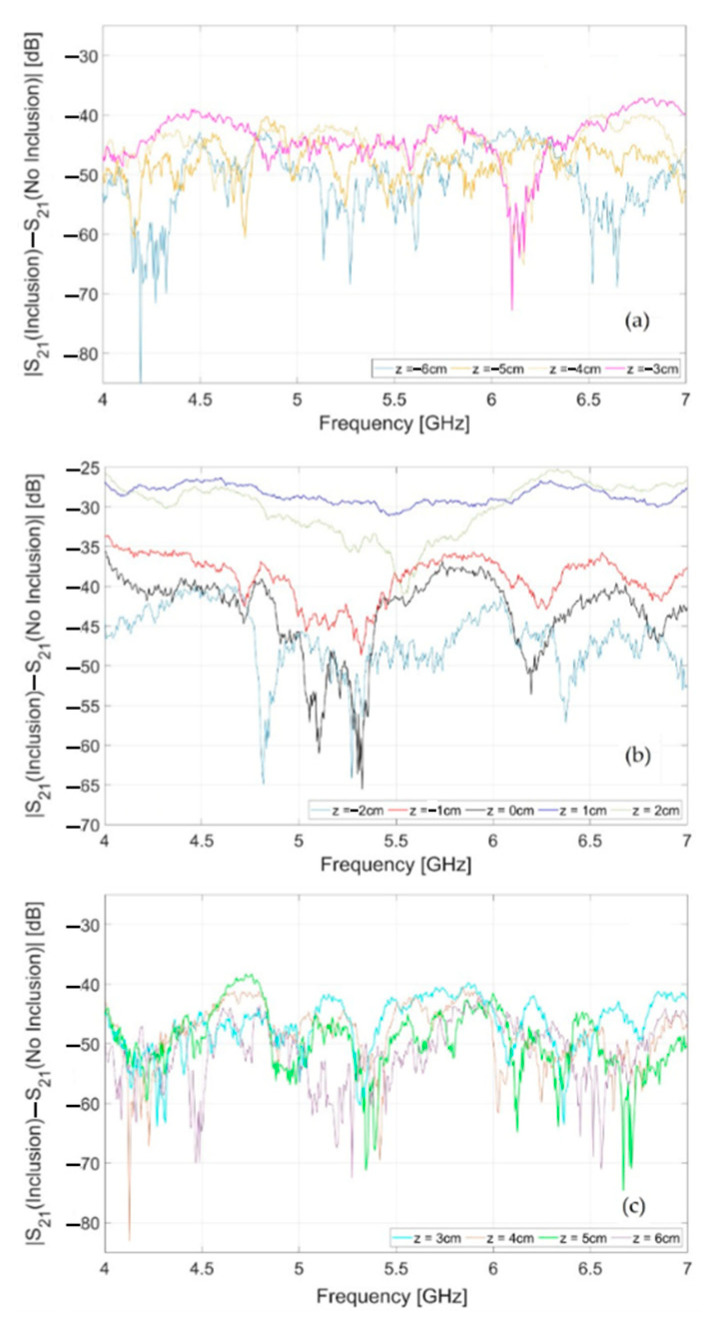
Magnitude (in dB), vs. frequency, of the difference between the transmission parameters measured when the OUT carries the inclusion and when the OUT is free of the inclusion. Each line refers to a different position of the OUT on the line: (**a**) *z_m_* = −6, −5, −4, −3 cm; (**b**) *z_m_* = −2, −1, 0, +1, +2 cm; (**c**) *z_m_* = +3, +4, +5, +6 cm.

For the sake of comparison, Figure 11 shows the magnitude (in dB) vs. the frequency of the difference between the transmission parameters measured when the OUT carries the inclusion and the ones measured when the OUT is free of the inclusion (reference data). Each line refers to a different position of the OUT on the line (panel (a): *z_m_* = −6, −5, −4, −3 cm; panel (b): *z_m_* = −2, −1, 0, +1, +2 cm; panel (c): *z_m_* = +3, +4, +5, +6 cm). These are the data to be provided as the input to the inversion algorithm implementing the RB approach. As can be seen, a remarkable difference is observed for certain positions (above −30 dB for z = +1 cm), revealing the presence of the inclusion in the OUT, which is absent in the reference object. Once again, this difference becomes more pronounced as the OUT, along with the inclusion, approaches the antennas’ aperture and becomes progressively less significant when the OUT moves away from the antennas.

The data in Figure 10 and Figure 11 were then processed through the respective inversion algorithms to provide an image of the inclusion.

**Figure 12:**
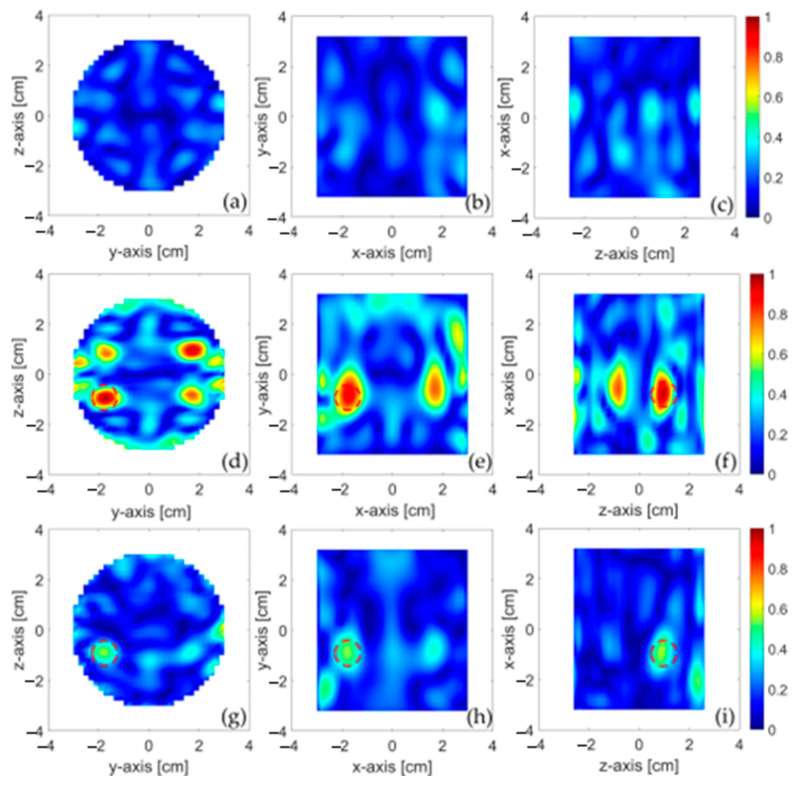
Reconstruction of the inclusion shown in three orthogonal cut planes crossing the center of the inclusion: (**a**–**c**) SB approach and OUT without the inclusion; (**d**–**f**) SB approach and OUT with the inclusion; (**g**–**i**) RB approach and OUT with the inclusion. The dashed circles in the panels represent the inclusion.

Figure 12 displays the reconstructions achieved for the magnitude of the electric contrast variation, say χ(*r*), due to the inclusion. Specifically, panels (a)–(c) show (in three different cut-planes) the reconstruction achieved through the SB approach, by processing the red data in Figure 10 (i.e., those relative to the OUT without the inclusion); panels (d)–(f) show the reconstructions obtained by processing the blue data in Figure 10 (i.e., those relative to the OUT with inclusion).

Finally, panels (g)–(i) present the reconstruction obtained by processing the data in Figure 11, through the RB approach. For the sake of comparison, all the maps were normalized to the maximum value, denoted as $max_{\underline{r},t}\left|\chi_{t}\left(\underline{r}\right)\right|$, where the subscript *t* indicates the type of MWI approach adopted to reconstruct χ(*r*), SB or RB.

From panels (a)–(c), it is noticeable that in the absence of the inclusion, a substantially flat map is returned with diffuse and weak spots, possibly due to the inaccurate manual positioning of the OUT at *z*_±*m*_, while well-defined and localized spots emerge in panels (d)–(f), indicating the presence of the inclusion. This confirms the effectiveness of the SB approach in providing reliable results for both possible situations: an OUT with and without an inclusion. An inherent inconvenience of this approach is that it does not yield a univocal image, but multiple images of the same inclusion: one at the actual position (indicated by the red dashed circles in Figure 12) and the three others at exactly specular positions with respect to the y = 0 and z = 0 planes (see panel (d)). The onset of these replicas is an unavoidable consequence of: I) the assumed symmetry along the z-axis, which does not allow for the determination of the actual position of the inclusion with respect to such an axis, and II) the reciprocity, which does not allow for the determination of the reciprocal position of the inclusion concerning the antennas (hence, with respect to the y-axis). From a practical point of view, this effect results from the reduced number of data available for the imaging, as half of them are used as reference data and half of them are equal for reciprocity. Nevertheless, given that the main aim of the system would be to discriminate between OUT with and without contaminants, the presence of the ghost images is not an actual shortcoming of the procedure. Additionally, the ambiguity can be overcome by building a more sophisticated MWI system and exploiting other symmetries, involving the reflection coefficients (for more details, the reader is referred to the numerical results in [20]), or rotating the OUT by 90 degrees and repeating the measurement.

Finally, the maps in panels (d)–(f) with those in panels (g)–(i), obtained with the RB approach, can be compared. As it can be observed, the RB approach also returns a spot just at the position of the inclusion (plus another less intense spot, arising at the specular positions with respect to the y-axis, again due to reciprocity), perfectly overlapping one of the spots in panels (d)–(f). The correspondence between the two reconstructions allows us to rule out that the result in (d)–(f) is an artifact, thus further confirming the effectiveness of the SB approach investigated in this paper. 

The results in Figure 10 and Figure 12 are encouraging, as they support the feasibility of the adopted reference-less imaging approach. However, it is also obvious that the actual feasibility of the proposed system for food quality monitoring is not yet demonstrated, given the size and the physical properties of the considered inclusion. 

To understand whether and to what extent it could be possible to detect and image targets of interest in the final application scenario, the numerical data reported in Figure 7 of [20], relative to a glass inclusion of 8 mm in size, can be exploited. In particular, by comparing the level of the numerical data in [20] with the level of the experimental data relative to the OUT without the inclusion (red lines in Figure 10), representing the “noise floor” of the system, the accuracy of the adopted experimental setup can be derived. From such a comparison, it follows that the “noise floor” level of the system (about −40 dB), is larger than the level of the estimated “useful” data for a piece of glass of 8 mm (about −50 dB). This means that, in the present conditions, performing an experiment to reveal an 8 mm glass inclusion would result in a useful signal completely embedded in the experimental “noise floor”. Accordingly, no imaging of the target could be possible. On the other hand, the comparison also shows that lowering the “noise floor” by just 20 dB would be sufficient to successfully record a useful signal and image realistic targets such as a glass splinter of 8 mm in size or even smaller. We expect that such a lowering is possible in consideration of the wide margin of improvements we can make to the system, as the ones listed below.

First of all, a significant improvement can be achieved by accurately implementing and assembling the system so as to remove all the sources of asymmetry (such as antennas misaligning, the probing of the OUT at not exactly specular positions, and so on).

Second, the system should be shielded from external disturbances, for instance, by enclosing it in a metallic box with the inner walls covered with absorbing panels. 

Third, the complexity of the system can be slightly increased, by considering more than one antenna for each side, so as to enable the collection of a larger amount of data. 

Finally, the image reconstruction algorithm can also be improved by more accurately modelling the experimental setup in order to better compute the Green’s function to be used in the inversion algorithm (see Appendix A).

These improvements to the experimental system will be the subject of future investigation. 

## 4. Conclusions

In this paper, we developed a preliminary proof of concept that involves the utilization of an MWI system based on two CLAVAs to detect contaminants in packaged food items. Additionally, we experimentally confirmed the effectiveness of an imaging approach that leverages the symmetries of both the MWI system and of the inspected object. In fact, as evidenced by the reconstructions and as it can be easily inferred from the scattering parameter trends, this experimental setup can detect the contaminant object inside the jar. From this point of view, the presence of “ghosts” can be sufficiently tolerated.

It is worth noting that imaging is not a crucial aspect in the food industry. This suggests that, although the imaging of contaminants can be useful, the primary focus is on the detection of anomalies or the contaminants themselves within food products. From this perspective, the proposed imaging method, such as the SB approach, appears to be sufficient to fulfill this task. The next step would be contaminant imaging. In this case, the SB approach alone is not enough, but an alternative would be to use it in conjunction with other methods, like the parallel symmetry plane (PSP) method presented in [20]. It’s important to emphasize that in the case under consideration, the parallel symmetry was intentionally not used because there is uncertainty about whether the jar is precisely centered between the two antennas, and therefore, the scattering parameters S_11_/S_22_ may not be symmetrical.

The study reports preliminary experimental results. This is due to several factors. First, the measurements were not conducted in a controlled environment, as an anechoic chamber was not used to eliminate potential interference. The system used consists of two antennas of the same type but not perfectly identical, resulting in slightly different scattering coefficients. Furthermore, the alignment of the system cannot be considered perfect, leading to some discrepancies in measurements when the jar’s position varied. To account for all these uncertainties, it was decided to use a sphere with a diameter larger than the contaminants one would expect in a jar of hazelnut cream. Nevertheless, it’s worth noting that the absence of a controlled environment may have its advantages. In an industrial setting, multiple reflections can occur during the process, potentially introducing interference that impacts the received signal at the antenna terminals.

Everything that has been performed so far represents a starting point; from here on, there are several things to do. First and foremost, it is necessary to build a dedicated measurement system with high symmetry, ensuring that the antennas comprising it exhibit consistent behavior. Additionally, it is necessary to repeat the measurements in a more controlled environment. This would allow this study to be replicated, not only with the proposed SB method but also with the PSP method. Furthermore, by eliminating the uncertainties of the system, it would be possible to use more realistic contaminants, such as half-centimeter-long fiberglass fibers.

## Figures and Tables

**Table 1:** Comparison of antennas designed for different sensing applications.

Ref.	Antenna	Frequency Band [GHz]	Dimensions [mm^2^]	Application
[18]	Slotted antenna with four star-shaped parasitic elements	3.8–10.1	29 × 26.6	Breast cancer detection
[21]	Circularly loaded Vivaldi antenna (CLAVA)	1.3–44	49.5 × 57	-
[24]	H-slot antenna with modified graded lens	0.7–1.7	100 × 120 (90)	Sensing applications
[25]	Narrow-beam Vivaldi antenna	2.5–8.5	55 × 82	Long distance detection
Present work	Circularly loaded Vivaldi antenna (CLAVA)	1.34–11	50 × 71.56	Food inspection

## Data Availability

The data presented in this study are available on request from the corresponding author.

## Abbreviations

CLAVA	Circularly Loaded Antipodal Vivaldi Antenna
MWI	MicroWave Imaging
OUT	Object Under Test
OSP	Orthogonal Symmetry Plane
PSP	Parallel Symmetry Plane
RB	Reference-Based
SB	Symmetry-Based
UWB	Ultrawideband
VNA	Vector Network Analyzer

## Appendix A

In this Appendix we recall the main aspects of the inversion procedure adopted in this work and proposed in [20].

Let us first consider the RB MWI approach. In this case, the data processed to create the image of the inclusion in Figure 12 are the differences, say ∆S, between the transmission parameters acquired for the OUT and the transmission parameters initially measured (and stored) for an identical but contaminant-free object assumed as reference [20]. Since it is assumed that the OUT and the reference object only differ by the possible presence of a contaminant, the ∆S data only account for the field scattered by the inclusion in the OUT, which, in turn, can be regarded as produced by an equivalent current distribution located in the OUT volume, say Ω. Now, if the inclusion can be modeled as a weak scatterer (i.e., it is electrically small), one can apply the Born approximation [26,27] and write: **J**_1(2)_(*r*) ≈ *i*ω*ε*_b_(*r*)χ(*r*)**E**_b1(2)_(*r*), *i* being the imaginary unity, ω the angular frequency, *ε*_b_(*r*) the complex permittivity of the reference object (or, equivalently, of the OUT without inclusion), **E**_b1(2)_(*r*) the electric field radiated by antenna 1 or 2 in the reference object (or, equivalently, in the OUT without inclusion) and χ(*r*) the variation/perturbation of the permittivity *ε*_b_(*r*) due to the possible presence of an inclusion in the OUT (the vector position *r* expresses the spatial dependence of the involved amounts, and belongs to the OUT volume, Ω). Note that, in the expression of **J**_1(2)_(*r*), *ε*_b_(*r*) and the field **E**_b1(2)_(*r*) are known being known the geometric and the electromagnetic properties of both the MWI system and the reference object (**E**_b1(2)_(*r*) can be computed through full-wave numerical simulation, as performed in Section 2.4). Therefore, the only unknown is the function χ(*r*), in Ω.

Now, by applying the reciprocity theorem to a volume including both the OUT and the antenna system, whose boundary cuts the antennas at the reference (i.e., the measurement) ports, one obtains (apart from an unessential constant factor):

(A1) $$\Delta S\left(z_{m},f_{n}\right)=\int_{\Omega }f_{n}E_{b1\left(2\right)}\left(z_{m},f_{n},\underline{r}\right)\cdot E_{b2\left(1\right)}\left(z_{m},f_{n},\underline{r}\right)\varepsilon_{b}\left(\underline{r}\right)\chi \left(\underline{r}\right)d\underline{r},$$

where the symbol “∙” expresses the dot product and the dependence on *z_m_* and *f_n_* indicates that (A1) holds for each probing frequency and position.

In the case of the SB MWI approach, being the approach still differential, the relation between the data ∆S and the unknown χ(*r*) is still given by (A1) with the difference that in this case ∆S = S_21_(*z_+__m_*, *f_n_*) − S_21_(*z_−__m_*, *f_n_*) and χ(*r*) is a replicated version of the actual contrast due to the symmetrization (see also Appendix A in [20]).

Since in (A1) the only unknown is the searched χ(*r*) in Ω, (A1) describes a linear integral operator between the data ∆S and the unknown χ(*r*), where the integral kernel (namely Green’s function) is:

(A2) $$K_{12}\left(z_{m},f_{n},\underline{r}\right)=f_{n}E_{b1}\left(z_{m},f_{n},\underline{r}\right)\cdot E_{b2}\left(z_{m},f_{n},\underline{r}\right)\varepsilon_{b}\left(\underline{r}\right),$$

(*K*_21_ = *K*_12_, for reciprocity). Note that the kernel *K*_21_ in (A2) is a not a singular function so that (A1) can be numerically solved by discretizing the searching domain Ω in Q electrically small cells so that in each of them one can assume *K*_21_(*z_m_*, *f_n_*, *r*) constant and equal to *K*_21_(*z_m_*, *f_n_*, *r_q_*), being *r_q_* position of the barycenter of the considered q-th cell of the mesh.

By doing so, (A1) becomes:

(A3) $$\Delta S\left(z_{m},f_{n}\right)=\sum_{q=1}^{Q}K_{12}\left(z_{m},f_{n},{\underline{r}}_{q}\right)\Omega_{q}\chi \left({\underline{r}}_{q}\right),$$

where Ω*q* is the volume of the q-th cell of the mesh and χ(*r_q_*) is the searched unknown evaluated at *r *=* r_q_*.

Equation (A3) can be expressed in the following matrix form:

(A4) $$\underline{\Delta S}={\underline{\underline{K}}}_{12}\;\underline{\underline{\Omega }}\;\cdot \;\underline{\chi }=\underline{\underline{A}}\cdot \underline{\chi },$$

where ∆S is a column vector of cardinality P = M*N representing the acquired data, $\underline{\underline{K}}$_21_ is a PxQ matrix whose the q-th column represents *K*_21_(*z_m_*, *f_n_*, *r_q_*), *m*,*n* = 1, …, P, $\underline{\underline{\Omega }}$ is a QxQ diagonal matrix whose the q-th entry of the main diagonal is *Ωq* and χ is the column vectors of the Q unknowns χ(*r_q_*), *q* = 1, …, Q (note that in our case Q > P).

According to (A4), the unknown vector χ can be estimated from the data vector by inverting the PxQ kernel matrix $\underline{\underline{A}}$ = $\underline{\underline{K}}$_21_∙

$$\underline{\underline{\Omega }}$$

. Usually, matrix $\underline{\underline{A}}$ is ill-conditioned, so its inversion requires a regularization procedure. Here, we adopted the truncated singular value decomposition (TSVD) scheme, relying on the result that $\underline{\underline{A}}$ can be expressed as: $\underline{\underline{A}}$ = $\underline{\underline{U}}$∙$\underline{\underline{\Sigma }}$∙$\underline{\underline{V}}$^†^, where $\underline{\underline{U}}$ and $\underline{\underline{V}}$ are PxP and PxQ unitary matrixes whose columns represent the left and right singular vectors of $\underline{\underline{A}}$, respectively (the symbol “†” denotes the transpose conjugation operation), while $\underline{\underline{\Sigma }}$ is a PxP diagonal matrix of ordered (in decreasing way) singular values of $\underline{\underline{A}}$, say σ_p_, p = 1, …, P. The TSVD approach consists of replacing, in (A4), the matrix $\underline{\underline{A}}$ with the matrix $\underline{\underline{A}}$_L_ = $\underline{\underline{U}}$_L_∙$\underline{\underline{\Sigma }}$_L_∙$\underline{\underline{V}}$_L_^†^ where $\underline{\underline{U}}$_L_ = $\underline{\underline{U}}$(1:P, 1:L), $\underline{\underline{\Sigma }}$_L_ = $\underline{\underline{\Sigma }}$(1:L, 1:L) and $\underline{\underline{V}}$_L_ = $\underline{\underline{V}}$(1:Q, 1:L), being L a truncation/regularization index (<P) set such that σ_L/_σ_1_ is larger than a prescribed threshold (set to 0.01 in the reconstructions of Figure 12, according to the measurement error). In other words, we disregard all the singular values, and the corresponding right singular vectors, of $\underline{\underline{A}}$ whose value, normalized to the largest singular value σ_1_, is smaller than the fixed threshold. The resulting matrix $\underline{\underline{A}}$_L_ is the best L-rank approximation of $\underline{\underline{A}}$ with a prescribed condition number σ_1/_σ_L_.

As a result, from (A4), with $\underline{\underline{A}}$_L_ in place of $\underline{\underline{A}}$, one can obtain an estimation of χ as follows:

(A5)
$$\underline{\tilde{\chi }}\;=\;{\underline{\underline{V}}}_{L}\cdot {\underline{\underline{\Sigma }}}_{L}^{-1}\cdot {\underline{\underline{U}}}_{L}^{+}\cdot \underline{\Delta S},$$

As a concluding remark, it is worth noting that the inversion Formula (A5) requires knowing the matrixes $\underline{\underline{U}}$_L_, $\underline{\underline{\Sigma }}$_L_ and $\underline{\underline{V}}$_L_ which, in turn, requires knowing matrix $\underline{\underline{A}}$, namely $\underline{\underline{K}}$_12_ in (A4). As described in Section 2.4, such a Green’s function matrix can be computed once a comprehensive numerical modeling of the overall system, i.e., MWI system plus OUT (without inclusion) has been constructed. In such sense, the inversion procedure in (A5) depends on the situation being considered.

## References

[1] Lau O.W., Wong S.K. (2000). Contamination in food from packaging material. J. Chromatogr. A.

[2] Nerín C., Aznar M., Carrizo D. (2016). Food contamination during food process. Trends Food Sci. Technol..

[3] Porep J.U., Kammerer D.R., Carle R. (2015). On-line application of near infrared (NIR) spectroscopy in food production. Trends Food Sci. Technol..

[4] Haff R.P., Toyofuku N. (2008). X-ray detection of defects and contaminants in the food industry. Sens. Instrum. Food Qual. Saf..

[5] Bick M., Sullivan P., Tilbrook D.L., Du J., Gnanarajan S., Leslie K.E., Foley C.P. (2015). A SQUID-based metal detector—Comparison to coil and X-ray systems. Supercond. Sci. Technol..

[6] Edwards M. (2004). Detecting Foreign Bodies in Food.

[7] Nikolova N.K. (2017). Introduction to Microwave Imaging.

[8] Wu Z., Wang H. (2017). Microwave tomography for industrial process imaging: Example applications and experimental results. IEEE Antennas Propag. Mag..

[9] Asefi M., Jeffrey I., LoVetri J., Gilmore C., Card P., Paliwal J. (2015). Grain bin monitoring via electromagnetic imaging. Comput. Electron. Agric..

[10] Garvin J., Abushakra F., Choffin Z., Shiver B., Gan Y., Kong L., Jeong N. (2023). Microwave imaging for watermelon maturity determination. Curr. Res. Food Sci..

[11] Vasquez J.A.T., Scapaticci R., Turvani G., Ricci M., Farina L., Litman A., Casu M., Crocco L., Vipiana F. (2020). Noninvasive inline food inspection via microwave imaging technology: An application example in the food industry. IEEE Antennas Propag. Mag..

[12] Tai T.C., Wu H.W., Hung C.Y., Wang Y.H. (2020). Food Security Sensing System Using a Waveguide Antenna Microwave Imaging through an Example of an Egg. Sensors.

[13] Augustin G., Denidni T.A. (2014). Ultrawideband Antennas for Microwave Imaging Systems.

[14] Zeni N., Bellizzi G., Crocco L., Cavagnaro M. A Compact Antipodal Vivaldi Antenna for Food Investigation. Proceedings of the 2023 17th European Conference on Antennas and Propagation (EuCAP).

[15] Farina L., Scapaticci R., Vasquez J.T., Rivero J., Litman A., Vipiana F. A feasibility study of a microwave imaging device for in-line food contamination monitoring. Proceedings of the 2019 13th European Conference on Antennas and Propagation (EuCAP).

[16] Darwish A., Ricci M., Zidane F., Vasquez J.A.T., Casu M.R., Lanteri J., Migliaccio C., Vipiana F. (2022). Physical Contamination Detection in Food Industry Using Microwave and Machine Learning. Electronics.

[17] Rafique U., Pisa S., Cicchetti R., Testa O., Cavagnaro M. (2022). Ultra-Wideband Antennas for Biomedical Imaging Applications: A Survey. Sensors.

[18] Zerrad F., Taouzari M., Makroum E.M., Aoufi J.E., Qanadli S., Karaaslan D.M., Abdullah Al-Gburi A.J., Zakaria Z. (2023). Microwave Imaging Approach for Breast Cancer Detection Using a Tapered Slot Antenna Loaded with Parasitic Components. Materials.

[19] Slimi M., Jmai B., Dinis H., Gharsallah A., Mateus P.M. (2022). Metamaterial Vivaldi Antenna Array for Breast Cancer Detection. Sensors.

[20] Zeni N., Crocco L., Cavagnaro M., Bellizzi G. (2023). A Simple Differential Microwave Imaging Approach for In-Line Inspection of Food Products. Sensors.

[21] Tayebi M., Dastranj A.A., Alighanbari A. UltraWideBand Antipodal Vivaldi Antenna with Tapered Triangular Corrugated Edges. Proceedings of the 2019 27th Iranian Conference on Electrical Engineering (ICEE).

[22] Wu Y., Lu J., Liu Y., Yang H. Modified design of the antipodal Vivaldi antenna. Proceedings of the ISAPE 2012.

[23] Mohammed B.J., Abbosh A.M., Mustafa S., Ireland D. (2014). Microwave System for Head Imaging. IEEE Trans. Instrum. Meas..

[24] Janani A.S., Darvazehban A., Rezaeieh S.A., Abbosh A.M. (2022). Focused Planar Electromagnetic Waves for Enhanced Near-Field Microwave Imaging with Verification Using Tapered Gradient-Index Lens Antenna. IEEE Access.

[25] Ren J., Fan H., Tang Q., Yu Z., Xiao Y., Zhou X. (2022). An Ultra-Wideband Vivaldi Antenna System for Long-Distance Electromagnetic Detection. Appl. Sci..

[26] Pastorino M. (2010). Microwave Imaging.

[27] Bertero M., Boccacci P. (1998). Introduction to Inverse Problems in Imaging.

